# MicroRNA-Detargeted Mengovirus for Oncolytic Virotherapy

**DOI:** 10.1128/JVI.02810-15

**Published:** 2016-03-28

**Authors:** Autumn J. Ruiz, Elizabeth M. Hadac, Rebecca A. Nace, Stephen J. Russell

**Affiliations:** Department of Molecular Medicine, Mayo Clinic College of Medicine, Rochester, Minnesota, USA; University of Iowa

## Abstract

Mengovirus, a member of the Picornaviridae family, has a broad cell tropism and can cause encephalitis and myocarditis in multiple mammalian species. Attenuation has been achieved by shortening the polycytidine tract in the 5′ noncoding region (NCR). A poly(C)-truncated strain of mengovirus, vMC_24_, resulted in significant tumor regression in immunocompetent BALB/c mice bearing syngeneic MPC-11 plasmacytomas, but the associated toxicities were unacceptable. To enhance its safety profile, microRNA target sequences complementary to miR-124 or miR-125 (enriched in nervous tissue), miR-133 and miR-208 (enriched in cardiac tissue), or miR-142 (control; enriched in hematopoietic tissues) were inserted into the vMC_24_ NCRs. The microRNA-detargeted viruses showed reduced replication and cell killing specifically in cells expressing the cognate microRNAs, but certain insertions additionally were associated with nonspecific suppression of viral fitness *in vivo. In vivo* toxicity testing confirmed that miR-124 targets within the 5′ NCR suppressed virus replication in the central nervous system while miR-133 and miR-208 targets in the 3′ NCR suppressed viral replication in cardiac tissue. A dual-detargeted virus named vMC_24_-NC, with miR-124 targets in the 5′ NCR and miR-133 plus miR-208 targets in the 3′ NCR, showed the suppression of replication in both nervous and cardiac tissues but retained full oncolytic potency when administered by intratumoral (10^6^ 50% tissue culture infectious doses [TCID_50_]) or intravenous (10^7^ to 10^8^ TCID_50_) injection into BALB/c mice bearing MPC-11 plasmacytomas. Overall survival of vMC_24_-NC-treated tumor-bearing mice was significantly improved compared to that of nontreated mice. MicroRNA-detargeted mengoviruses offer a promising oncolytic virotherapy platform that merits further development for clinical translation.

**IMPORTANCE** The clinical potential of oncolytic virotherapy for cancer treatment has been well demonstrated, justifying the continued development of novel oncolytic viruses with enhanced potency. Here, we introduce mengovirus as a novel oncolytic agent. Mengovirus is appealing as an oncolytic virotherapy platform because of its small size, simple genome structure, rapid replication cycle, and broad cell/species tropism. However, mengovirus can cause encephalomyelitis and myocarditis. It can be partially attenuated by shortening the poly(C) tract in the 5′ NCR but remains capable of damaging cardiac and nervous tissue. Here, we further enhanced the safety profile of a poly(C)-truncated mengovirus by incorporating muscle- and neuron-specific microRNA target sequences into the viral genome. This dual-detargeted virus has reduced pathogenesis but retained potent oncolytic activity. Our data show that microRNA targeting can be used to further increase the safety of an attenuated mengovirus, providing a basis for its development as an oncolytic platform.

## INTRODUCTION

Oncolytic virotherapy employs naturally occurring or engineered viruses that preferentially infect and kill tumor cells and stimulate both innate and tumor-specific adaptive immune responses (1). Clinical evaluation of oncolytic virotherapy has yielded promising results; however, translational success can be hindered by a lack of relevant animal models for preclinical evaluation, preexisting antiviral immunity, suboptimal viral extravasation in solid and disseminated cancers, and a requirement for extremely high viral doses for effective systemic therapy constrained by manufacturing limitations and necessitating highly attenuated viruses (2–4).

Picornaviruses have emerged as promising oncolytic agents, with clinical trials either under way or completed for coxsackievirus type A21, Seneca Valley virus, a poliovirus-rhinovirus chimera (PVSRIPO), and enteric cytopathic human orphan type 7 virus, which has been approved for melanoma therapy in Latvia (3, 5–7). Picornaviruses are rapidly replicating, small, nonenveloped, single-stranded, positive-sense RNA viruses. Certain picornaviral infections can generate sustained, high-level viremia following replication at a primary site of infection, which may prove advantageous for treating disseminated or metastatic cancers that require an increased viremic threshold and duration. Picornavirus genomes are between 7 and 9 kb in length and are small enough to be inserted within a single plasmid, allowing for simplified genetic manipulation and virus production. Additionally, small viral particles (∼27 to 30 nm) are more likely to extravasate from tumor blood vessels (4). Moreover, there are several picornaviruses for which human seropositivity is low and infection typically is asymptomatic. Some of these viruses also can be highly antigenic and induce potent humoral and cellular immune responses to mediate indirect killing of tumor cells (8–12).

Mengo encephalomyocarditis virus (mengovirus) is a member of the Cardiovirus genus in the Picornaviridae family. It is a zoonotic pathogen and is serologically related to encephalomyocarditis virus (EMCV). While rodents are the most common natural host, mengovirus can infect several mammals, including nonhuman primates, pigs, and elephants, and also can infect birds without extensive adaptation (13). This is advantageous because it broadens the range of cancers potentially susceptible to mengovirus therapy, and the therapeutic efficacy and safety profile of the virus can undergo extensive preclinical evaluation in relevant animal models with intact or suppressed immune systems. The sporadic isolation of mengovirus from sick humans has been documented; however, no association between the prevalence of virus or antiviral antibodies and the incidence of disease has been established (14). Overall, mengovirus infection in humans appears to be generally asymptomatic and to exhibit very low seroprevalence (15).

Mengovirus infection is acquired through the ingestion of contaminated feed, drinking water, or infected animal carcasses. It is a lytic virus that results in necrotic cell death following virus-mediated inhibition of apoptosis. Depending on the dose, route of administration, strain, animal species, and immunocompetence of the host, the M strain of mengovirus can cause severe meningoencephalomyelitis and/or myocarditis manifesting as flaccid hind-limb paralysis (HLP), labored breathing, and/or sudden death (16, 17). A major determinant of virulence is a homopolymeric polycytidine tract (pCT) within the 5′ noncoding region (NCR). Live attenuated strains of mengovirus that contain a truncated pCT (C_13_UC_10_) or a pCT deletion (vMC_24_ and vMC_0_, respectively) have markedly reduced pathogenicity in various mammals compared to the wild-type virus (pCT, C_44_UC_10_) (18, 19). Both attenuated strains have been used successfully to vaccinate wild and domesticated animal species against EMCV-like viruses with no adverse reactions (20, 21). However, in preliminary studies designed to assess the oncolytic potential of vMC_24_, we determined that this attenuated virus maintains the potential for the development of neurotropic and cardiotropic toxicities following the administration of doses required for anticancer activity. Therefore, the aim of this study was to eliminate these toxicities and generate a safe and potent oncolytic virus that could be used to treat a broad range of cancers.

MicroRNA (miRNA) targeting has been well validated as an approach to eliminate unwanted picornavirus tropisms (22–24). miRNAs are short (22 to 25 bases), noncoding RNAs that mediate the posttranscriptional regulation of gene expression in multicellular eukaryotes. miRNAs bind complementary sequences present in target mRNAs, resulting in either the degradation/destabilization or translational repression of the transcripts. The insertion of miRNA target (miRT) sequences into viral genomes has proven an effective method of regulating the tissue tropism of many oncolytic viruses (25). Picornaviruses, including poliovirus and coxsackievirus A21, have proven exceptionally sensitive to miRNA targeting because of the positive-sense-oriented genome and their completely cytoplasmic replication cycle (22, 23, 25). Here, we report that target sequences for miRNAs enriched within nervous and cardiac tissues can be incorporated into specific sites in both the 5′ and 3′ NCRs of mengovirus without compromising replication in cells that do not express the respective cognate miRNAs while at the same time blocking replication in cells that do express the cognate miRNAs. Treatment of immunocompetent mice bearing subcutaneous (s.c.) multiple myeloma tumors using a dual-detargeted virus incorporating both cardio-specific and neuron-specific miRTs resulted in tumor regression and significantly prolonged survival of treated mice without significant virus-mediated toxicity.

## MATERIALS AND METHODS

### Cells.

MPC-11 (ATCC CCL-167), EMT-6 (ATCC CRL-2755), B16-F10 (ATCC CRL-6475), A20 (ATCC TIB-208), EL4 (ATCC TIB-39), Hepa 1-6 (ATCC CRL-1830), H1HeLa (ATCC CRL-1958), RAW 264.7 (ATCC TIB-71), U266 (ATCC TIB-196), RPMI 8226 (ATCC CCL-155), and ARH77 (ATCC CRL-1621) cell lines were purchased from the American Type Culture Collection (ATCC; Manassas, VA). Other human myeloma cell lines were obtained at the Mayo Clinic courtesy of Diane Jelinek (Kas6/1 and JJN-3; Rochester, MN) or Rafael Fonseca (MM-1; Scottsdale, AZ). The 5TGM1 cell line, obtained from Babatunde Oyajobi (UT Health Sciences Center, San Antonio, TX), was grown in Iscove's modified Dulbecco's medium (IMDM; 12440; Life Technologies, NY) supplemented with 10% fetal bovine serum (FBS). MPC-11 and EL4 cells were grown in Dulbecco's modified Eagle's medium (DMEM; SH30022.01; Thermo Scientific, MA) supplemented with 10% horse serum. A20 cells were grown in RPMI 1640 medium (10-040-CV; Thermo Scientific, MA) with 10% FBS and 0.05 mM 2-mercaptoethanol. U266, RPMI 8226, ARH77, JJN-3, and MM-1 cells were grown in RPMI 1640 with 10% FBS. Kas6/1 cells were grown in RPMI 1640 with 10% FBS and 1 ng/ml interleukin-6 (IL-6). All other cell lines were grown in DMEM with 10% FBS. All cell lines additionally were supplemented with 100 U/ml penicillin and 100 μg/ml streptomycin and grown at 37°C in 5% CO_2_. The H1HeLa, RPMI 8226, and ARH77 cells were authenticated by the ATCC using short tandem repeat DNA profiling. The ATCC routinely tests morphology, karyotype, and species. We performed no further authentication of the cell lines. 5TGM1 cells were not authenticated, but specific IgG2b paraprotein secretion was verified by enzyme-linked immunosorbent assay (ELISA). All cells routinely tested negative for mycoplasma contamination.

### pMC_24_-miRT plasmids.

The pF/R-wt and RZ-pMwt plasmids were kind gifts from Ann C. Palmenberg (University of Wisconsin, Department of Biochemistry and the Institute for Molecular Virology), and their construction has been described already (26, 27). The cytomegalovirus (CMV) promoter from pF/R-wt was cloned into RZ-pMwt to obtain pCMV-RZ-Mwt. A double-stranded DNA (dsDNA) fragment containing nucleotides (nt) 43 to 105, C_13_UC_10_, and nt 203 to 335 (numbering based on RZ-pMwt) was synthesized and cloned into pUC57 (GenScript, Piscataway, NJ). The fragment was subcloned into pCMV-RZ-Mwt at EcoRV and AvrII sites (pMC_24_). miRNA sequences were obtained from Sanger Institute miRBase. Two copy inserts were cloned into pMC_24_ using splice-overlap extension (SOE) PCR. Two complementary single-stranded oligonucleotides encoding 3 or 4 copies of miRNA target inserts flanked by NheI or XhoI restriction site overhang sequences were annealed in T4 DNA ligase buffer (B0202S; NEB, MA) by heating to 85°C and slowly cooling to 25°C. NheI and XhoI sites were cloned upstream of pCT and into the 3′ NCR at position 7617 of pMC_24_, respectively, using SOE PCR, allowing the insertion of annealed oligonucleotides. Dual-detargeted pMC_24_ was generated by ligating together fragments from individually targeted plasmids at EcoRV and AvrII sites. The integrity of the targets was verified by sequencing.

### Recombinant vMC_24_ viruses.

A total of 4 × 10^5^ H1HeLa cells were seeded per well into 6-well plates and incubated at 37°C in 5% CO_2_ overnight. A total of 2.5 μg of pMC_24_ or miRT-pMC_24_ was transfected per well using Mirus *Trans*IT-2020 transfection reagent (MIR 5400; Mirus Bio LLC, Madison, WI) according to the manufacturer's instructions. Once cytopathic effects (CPE) were apparent (24 to 72 h), the cells were scraped into the supernatant for sample collection. Samples were subjected to three freeze-thaw cycles, the cellular debris was removed by centrifugation, and the cleared lysate was filtered through a 0.22-μm filter and passaged onto fresh H1HeLa cells. For *in vivo* studies with intravenous administration, vMC_24_-NC filtered viral supernatant was layered onto a 10% sucrose cushion and concentrated through ultracentrifugation at 27,000 rpm for 1 h at 4°C. The viral pellet was resuspended in 1× phosphate-buffered saline (PBS). Viral RNA was isolated from all virus stocks using a QiaAmp viral RNA minikit (52904; Qiagen, CA) according to the manufacturer's instructions. Regions containing the miRT inserts were amplified using the Titan one-tube reverse transcription-PCR (RT-PCR) system (11855476001; Roche Applied Science, IN), and the integrity of the inserts was verified via sequencing.

### Virus titration.

A total of 1 × 10^4^ H1HeLa cells were seeded per well into 96-well plates and grown at 37°C in 5% CO_2_. At 24 h, 10-fold serial dilutions of each virus stock were made and 100 μl of each dilution was added to each of eight replicate wells. The cells were incubated at 37°C in 5% CO_2_ for 72 h. The cells were visually assessed for CPE and scored as positive or negative. The 50% tissue culture infectious dose (TCID_50_) per milliliter was calculated using the Spearman and Kärber equation.

### Cell proliferation assays.

3-(4,5-dimethylthiazolyl-2)-2,5-Diphenyltetrazolium bromide (MTT) (30-1010K; ATCC, VA) analysis was used to measure unmodified or miRT-vMC_24_ cytotoxicity in all cell lines. Cells were infected for 2 h at 37°C in 5% CO_2_, followed by replacement with fresh complete growth medium. Cell viability was analyzed 72 h later, and all samples were normalized to mock-infected cells.

### MicroRNA targeting assays.

miRIDIAN miRNA mimics and a negative-control mimic corresponding to a Caenorhabditis elegans miRNA were purchased from Dharmacon (GE Dharmacon, CO). miRNA mimics were transfected into H1HeLa cells using *Trans*IT-mRNA transfection reagent at a concentration of 200 nM. Six hours later, cells were infected with unmodified virus or miRT-vMC_24_ at an MOI of 0.2. At 24 h postinfection, the supernatants were collected and cleared for titration and the cells assayed for proliferation as described above.

### Single-step growth curves.

H1HeLa or RAW 264.7 cells were infected at an MOI of 3 for 2 h at 37°C in 5% CO_2_, followed by removal of unincorporated virus and addition of fresh complete growth medium. At 2, 4, 6, 8, 10, 24, and 48 h postinfection, cells were scraped into the supernatant and samples frozen at −80°C. Once all samples had been collected, they underwent three freeze-thaw cycles and the cellular debris were removed through centrifugation. Cleared lysates were titrated on H1HeLa cells as described above.

### Genetic stability of miRNA targets.

H1HeLa cells initially were infected with vMC_24_-NC at an MOI of 0.1, and when CPE was complete the samples were collected. MPC-11 cells initially were infected at an MOI of 10, and samples were collected 24 h later. All samples were subjected to three freeze-thaw cycles, and the lysates were clarified by centrifugation and filtered through a 0.22-μm filter. Virus in clarified lysates was passaged serially in H1HeLa cells 10 times, each time using 1 volume of clarified lysate to 3 volumes of fresh media, or in MPC-11 cells 10 times, each using 1 volume of clarified lysate to 2 volumes of fresh media to prepare high-MOI inocula. Viral RNA was isolated from the cleared lysates with a QIAamp viral RNA minikit (Qiagen, CA) according to the manufacturer's instructions. cDNA was synthesized using Superscript III first-strand synthesis supermix (18080-400; Life Technologies, NY) according to the manufacturer's instructions. Regions containing the microRNA target insert sequences were amplified using an Expand high-fidelity PCR kit (04738250001; Roche Applied Science, IN) according to the manufacturer's instructions, modified only by adding dimethyl sulfoxide (DMSO) to each reaction mix. For H1HeLa serial-passage samples, amplicons were ligated into a pGEM-T Easy cloning vector (A1360; Promega, WI) and at least 15 individual clones sequenced.

### Animal experiments.

The Mayo Clinic Institutional Animal Care and Use Committee approved all animal studies. For intracranial (i.c.) administration studies, 4- to 5-week-old female C57BL/6 mice were purchased from Jackson Laboratories (Bar Harbor, ME). All viruses were diluted in Opti-MEM I reduced serum medium (31985-070; Life Technologies, NY), and 1 × 10^5^ TCID_50_ was delivered per mouse. All mice were euthanized 4 days later, and brain, spinal cord, and heart tissues were harvested and flash frozen for virus titration.

For intratumoral (i.t.) administration studies, 5 × 10^6^ washed MPC-11 cells were implanted subcutaneously into 4- to 5-week-old female BALB/c mice (Jackson Laboratories). When tumors reached an average diameter of 0.5 cm, mice were treated with Opti-MEM or 1 × 10^6^ TCID_50_ vMC_24_ or vMC_24_-miRT diluted in Opti-MEM.

For intravenous (i.v.) administration studies, 5 × 10^6^ washed MPC-11 cells were implanted subcutaneously into 4- to 5-week-old female BALB/c mice (Jackson Laboratories). When tumors reached an average diameter of 0.5 cm, mice were treated with Opti-MEM, 1 × 10^7^ TCID_50_ vMC_24_, 1 × 10^7^ TCID_50_ vMC_24_-NC, or 1 × 10^8^ TCID_50_ vMC_24_-NC through the tail vein. At 4 days posttreatment with vMC_24_-NC, four mice per group (including controls) were euthanized. Tissues were harvested and immediately flash frozen for virus titration and miRNA target genetic stability analysis.

All tumor-bearing mice were observed and weighed, and tumor size was measured daily using a handheld caliper. Mice were anesthetized through the inhalation of isoflurane, and blood was obtained through cardiac puncture at the time of euthanasia. Harvested tissues were immediately sectioned into a vessel containing the appropriate preservative and placed at the appropriate temperature.

### Plasma virus titration.

Mice were anesthetized through the inhalation of isoflurane, and blood was collected from the submandibular vein in a BD Microtainer tube with a lithium heparin and plasma separator (365958; BD Biosciences). Plasma was separated by centrifugation according to the manufacturer's instructions. Viral loads were determined by titrating on H1HeLa cells as described above, and the TCID_50_ per milliliter was calculated.

### Tissue virus titration and miRNA target genetic stability analysis.

Whole organs (single lobe for liver) from i.c.- or i.v.-injected mice harvested 4 days after virus administration were weighed and homogenized using Kimble-Chase Kontes pellet pestles (K749520; Fisher Scientific) and suspended in a total volume of 1 ml DMEM. Tissue suspensions were subjected to three freeze-thaw cycles and centrifuged. The cleared tissue lysates were titrated on H1HeLa cells as described above, and the TCID_50_ per milligram of tissue was calculated. Viral RNA was isolated from cleared tissue lysates with a QiaAmp viral RNA minikit (Qiagen, CA) according to the manufacturer's instructions. cDNA was synthesized using Superscript III first-strand synthesis supermix (18080-400; Life Technologies, NY) according to the manufacturer's instructions. Regions containing the microRNA target insert sequences were amplified using an Expand high-fidelity PCR kit (04738250001; Roche Applied Science, IN) according to the manufacturer's instructions, which were modified only by adding DMSO to each reaction.

Organs harvested from all other tumor-bearing mice were segmented and one section flash frozen. These sections were processed as described above to generate tissue suspensions for titration.

### Histopathology analysis of tissues.

Tissues harvested at the time of euthanasia were segmented, and one section was stored in 10% neutral buffered formalin. Histological and pathological analyses of tissues were performed by the Mayo Clinic Scottsdale Research Histology Core.

### Statistical analysis.

GraphPad Prism software, version 5.0a (GraphPad Software, La Jolla, CA), was used for data analysis and graphical representations. Two-tailed unpaired Student *t* tests with Welch's correction (for unequal variances) were used for statistical analysis of miRNA mimic targeting assays, and *P* < 0.01 was considered statistically significant. Survival curves were plotted according to the Kaplan-Meier method, and the survival rates across treatment groups were compared using log-rank tests.

## RESULTS

### vMC_24_ has oncolytic activity *in vitro*.

**FIG 1:**
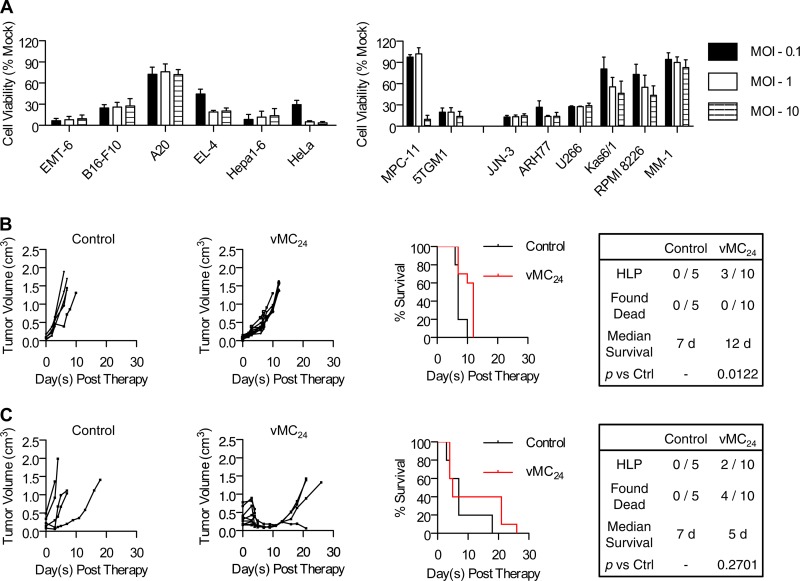
vMC_24_ has oncolytic activity against a broad range of cancer cell lines and exhibits potent oncolytic activity *in vivo*, but it causes lethal toxicity. (A) Cytotoxicity of vMC_24_ against a panel of human and murine cancer cell lines. Data are presented as mean percent cell viability relative to that of mock-infected cells at 72 h postinfection from duplicate or triplicate experiments ± standard deviations. (B and C) BALB/c mice bearing s.c. MPC-11 tumors were treated with a single injection of vMC_24_ i.v. (1 × 10^7^ TCID_50_) (B) or i.t. (1 × 10^6^ TCID_50_) (C), and tumor burden was monitored by calculating tumor volume versus time using repeated caliper measurements. Overall survival of control Opti-MEM-treated and vMC_24_-treated mice was assessed using Kaplan-Meier survival curves. The tabulation of the causes of death/euthanasia other than tumor burden in all treated mice, median survival, and significance of overall survival benefit in vMC_24_-treated mice versus control mice based on log-rank statistics are shown.

The cytolytic activity of vMC_24_ initially was evaluated against a panel of human and murine cancer cell lines, including two murine and six human multiple myeloma cell lines. Cells were infected with vMC_24_ at various multiplicities of infection (MOI), and the viability of the cells was determined at 72 h postinfection (Fig. 1A). Of the non-multiple myeloma cell lines tested, the murine B cell lymphoma A20 cell line proved most resistant to vMC_24_, with an ∼30% loss in viability. All other non-myeloma cell lines were highly susceptible to infection. The majority of the multiple myeloma cell lines were susceptible to vMC_24_ infection. The murine MPC-11 and human MM-1 cell lines demonstrated the most resistance. vMC_24_ infection of MM-1 cells resulted in an ∼11% loss in viability, and significant loss in the MPC-11 line cell viability was observed only at high MOI. These results demonstrate the potential of vMC_24_ as a broadly applicable oncolytic agent.

### vMC_24_ has potent oncolytic activity *in vivo* but causes lethal toxicity.

The oncolytic activity of vMC_24_ was analyzed *in vivo* against the murine multiple myeloma MPC-11 syngeneic mouse tumor model. The MPC-11 cancer cell line proved more resistant than the other cancer cell lines tested, providing a stringent and clinically relevant *in vivo* model for analyzing the therapeutic efficacy and toxicity of vMC_24_. Additionally, this cell line was chosen because it can be used for preclinical evaluation of solid and systemic tumor treatment in immunocompetent mice. Immunocompetent BALB/c mice bearing s.c. MPC-11 tumors were treated with a single intravenous (i.v.) dose of 1 × 10^7^ TCID_50_ vMC_24_ or a single intratumoral (i.t.) dose of 1 × 10^6^ TCID_50_ vMC_24_. Tumor volume and overall survival readouts were used to evaluate therapeutic efficacy. As shown in Fig. 1B and C, all control-treated mice exhibited continuous rapid tumor growth. Mice treated i.v. with vMC_24_ exhibited a delay in tumor growth; however, no significant regression was observed (Fig. 1B). Three of these mice developed hind-limb paralysis (HLP) 7 days after vMC_24_ treatment, and the remaining seven mice all were euthanized due to high tumor burden between days 10 and 12 after therapy. In contrast, all vMC_24_ IT-treated mice had decreased tumor volumes within 5 days, indicative of direct tumor lysis by the virus. Between days 4 and 5 posttherapy, two mice developed HLP and four mice were found dead. One additional mouse had to be euthanized 21 days posttherapy due to wasting. All mice were checked daily for signs of failing health, and the animals that were found dead each had appeared well the day prior to death. Therefore, myocarditis leading to the sudden onset of cardiac arrhythmia was reported to be the cause of death. Although vMC_24_ treatment resulted in tumor regression following i.t. administration, no significant increase in survival was observed due to viral toxicity (Fig. 1C).

### vMC_24_ tolerates microRNA target inserts in the 5′ and 3′ NCRs.

**FIG 2:**
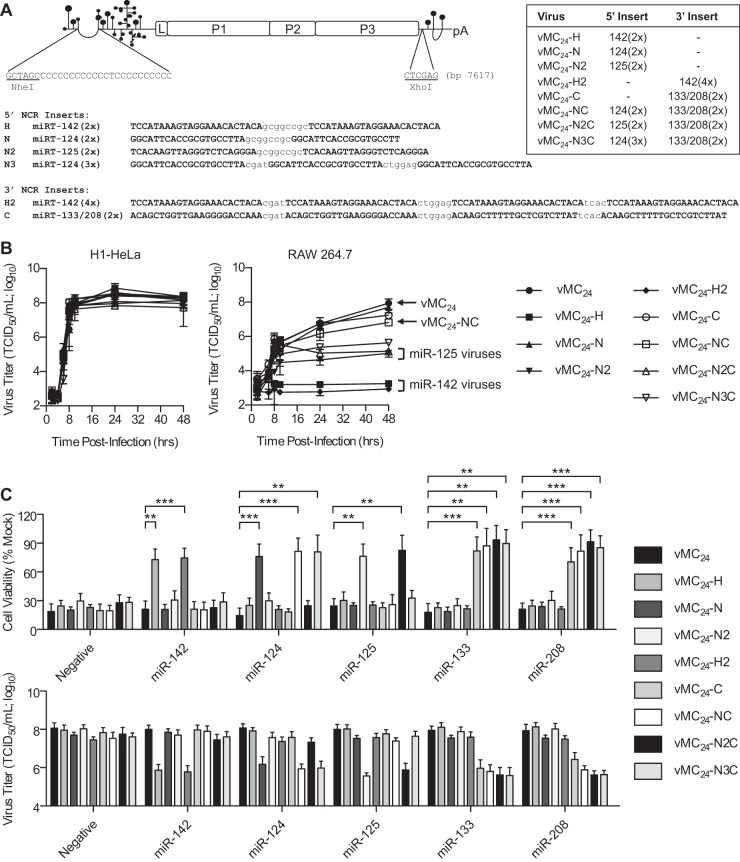
miRNA targeting regulates vMC_24_ tropism without altering replication kinetics. (A) Schematic representation of the mengovirus genome and location and sequence of miRNA target inserts. miRT virus designations and their respective target inserts and location are shown. (B) Replication kinetics of miRT-vMC_24_ viruses were evaluated using single-step growth curve analysis (MOI, 3) in H1HeLa cells and RAW 264.7 macrophages. All experiments were repeated at least four times, and data are represented as mean viral titers ± standard deviations. (C, top) H1HeLa cells transfected with individual miRNA mimics were infected with vMC_24_ or miRT-vMC_24_ at an MOI of 0.2, and cell viability was determined at 24 h postinfection. (Bottom) Viral titers in the supernatants of the treated cells also were determined at 24 h postinfection. All experiments were repeated at least four times, and the data are represented as mean viability or viral titer ± standard deviations. A *P* value of <0.01 was considered significant (**, *P* < 0.01; ***, *P* < 0.001).

Many miRNAs are enriched within specific tissues, and tumor cells often exhibit aberrant miRNA expression profiles. In order to control both the neurotropism and cardiotropism of vMC_24_, thereby eliminating the risk of viral encephalomyelitis and myocarditis, we investigated the targeting of miRNAs known to be abundantly expressed in the brain and the heart. miR-125b is ubiquitously expressed in all cell lineages of the brain, whereas miR-124 is highly enriched in neurons (28–33). Additionally, miR-133b and miR-208a are enriched within cardiomyocytes of adult mice and humans (29, 34–36). As a control, we employed miR-142, which is exclusively expressed in cells of hematopoietic lineage (37, 38). The pCT in cardioviruses and apthoviruses can range anywhere from 60 to 400 bases in length (39). Additionally, the 15 bases directly 5′ of the pCT in mengovirus are dispensable for growth and have been hypothesized to serve as a vestigial spacer between the 5′ pseudoknots and the pCT (40). Based on these analyses, we predicted that miRT sequences would be well tolerated directly preceding the truncated pCT of vMC_24_. Additional studies predicted three conserved stem-loops in the 3′ NCR of mengovirus (41). Based on these data, we hypothesized that miRT sequences would be best tolerated in the region 5′ of the first stem-loop with nonconserved structure and function. Therefore, we generated detargeted viruses by inserting two tandem copies of miR-124, miR-125b, or either miR-142-3p target sequences immediately preceding the pCT in the 5′ NCR or two copies each of miR-133b and miR-208a [133/208(2×)] target sequences or four copies of miR-142-3p target sequences in the 3′ NCR of vMC_24_. We also generated detargeted viruses containing two copies each of either miR-124 or miR-125b target sequences prior to the pCT in the 5′ NCR and the 133/208(2×) insert in the 3′ NCR. We also constructed a virus with three copies of the miR-124 targets upstream of the pCT in the 5′ NCR and the 133/208(2×) insert in the 3′ NCR. A schematic representation of these miRT-vMC_24_ constructs and their corresponding identifications are shown in Fig. 2A. The transfection of plasmid DNA expressing each of the modified viral genomes into H1HeLa cells resulted in infectious recombinant miRT-vMC_24_ virus recovery with sequence-verified microRNA target inserts.

### miRNAs regulate tropism of vMC_24_ encoding complementary target sequences.

The replication of miRT viruses was compared to that of unmodified vMC_24_ using single-step growth curve analysis (Fig. 2B) in H1HeLa cells, which do not express any of the corresponding miRNAs, and in RAW 264.7 macrophages, which express high levels of miR-142 and intermediate levels of miR-125b (37, 38, 42). All miRT viruses replicated at rates similar to those of unmodified vMC_24_ in H1HeLa cells. However, in RAW 264.7 macrophages, viruses encoding either miR-125b or miR-142 target sequence replicated with diminished kinetics or did not replicate at all, resulting in significantly lower peak viral yields. These data indicate that the engineered microRNA target inserts do not affect the replication kinetics of the virus and that naturally occurring miRNAs can interact with the target sequences.

The specificity of miRNA-mediated regulation of viral tropism was analyzed by measuring cell viability and viral replication in H1HeLa cells transfected with complementary or noncomplementary synthetic miRNA mimics. An miRNA mimic corresponding to a Caenorhabditis elegans miRNA absent from mammalian cells was used as a negative control. Six hours posttransfection with miRNA mimics, the cells were infected with vMC_24_ or miRT-vMC_24_ at an MOI of 0.2. Twenty-four hours later, the viability of the cells was assessed and the virus titer in the supernatant was determined. All miRT-vMC_24_ variants had significantly reduced cytotoxicity and replication (Fig. 2C) in cells transfected with the complementary miRNA but not in those expressing noncomplementary miRNAs. The protective activity of each miRT was similar, and no significant differences were observed in comparisons of viruses with three or four copies of miRNA targets to those with only two.

### MicroRNA targeting restricts mengovirus vMC_24_ tropism *in vivo*, ameliorating its cardio- and neurotoxicity.

**FIG 3:**
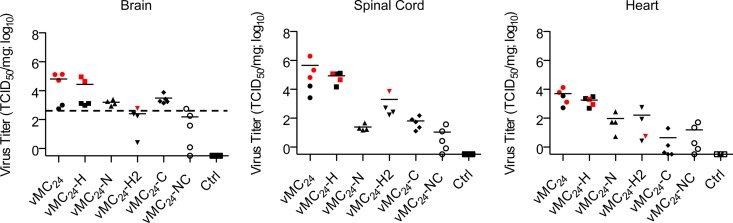
miRNA targeting regulates viral tropism *in vivo* and ameliorates cardiotoxicity and neurotoxicity. C57BL/6 mice were injected i.c. with Opti-MEM (*n* = 3) or 1 × 10^5^ TCID_50_ of vMC_24_-H (*n* = 5), vMC_24_-N (*n* = 4), vMC_24_-H2 (*n* = 4), vMC_24_-C (*n* = 5), or vMC_24_-NC (*n* = 5). Four days postinjection, all mice were euthanized and the infectious viral titers within the brain, spinal cord, and heart determined. Red symbols denote mice that developed HLP. The dotted line in brain tissue titration data represents the theoretical maximum input virus recovery and is based on the average brain weight of all mice analyzed. Individual viral titers are plotted along with the mean viral load (solid line).

Five miRT viruses were evaluated *in vivo*, vMC_24_-H, vMC_24_-N, vMC_24_-H2, vMC_24_-C, and vMC_24_-NC. The miRNA-mediated control of virus replication and toxicity in nervous tissue was investigated by injecting 1 × 10^5^ TCID_50_ of either vMC_24_ or miRT-vMC_24_ intracranially into C57BL/6 mice, which are highly susceptible to vMC_24_ central nervous system (CNS) infection (19). All mice were euthanized 4 days postinjection, and the viral load within the brain, spinal cord, and heart were determined (Fig. 3). vMC_24_-injected mice had mean viral titers between 5 × 10^3^ and 5 × 10^5^ TCID_50_ per mg in all three tissues. Additionally, the three mice with the highest viral loads in the brain and spinal cord developed HLP. vMC_24_-H-injected mice had similar viral titers, and again the two mice with the highest viral loads in the brain developed HLP. Mice injected with vMC_24_-N had reduced viral loads in all three tissues, and no clinical signs of neurotoxicity were observed. Unexpectedly, mice injected with vMC_24_-H2 or vMC_24_-C also had reduced mean viral loads in all three tissues, suggesting that either the placement of the insert can control viral replication *in vivo* or that there are low to intermediate levels of miR-142, miR-133, or miR-208 present regulating viral tropism. One mouse injected with vMC_24_-H2 developed HLP and had elevated virus titers in the spinal cord compared to those of the other mice in the group. Mice injected with vMC_24_-C had reduced viral titers in the heart compared to those of vMC_24_-H2-injected mice, indicating miRNA-mediated control. Finally, mice treated with vMC_24_-NC had greatly reduced viral loads in all three tissues compared to those of all other virus-injected mice, indicative of further suppression of viral replication attributed to miRNA targeting.

### Genetic stability of miRNA target sequences *in vitro*.

**FIG 4:**
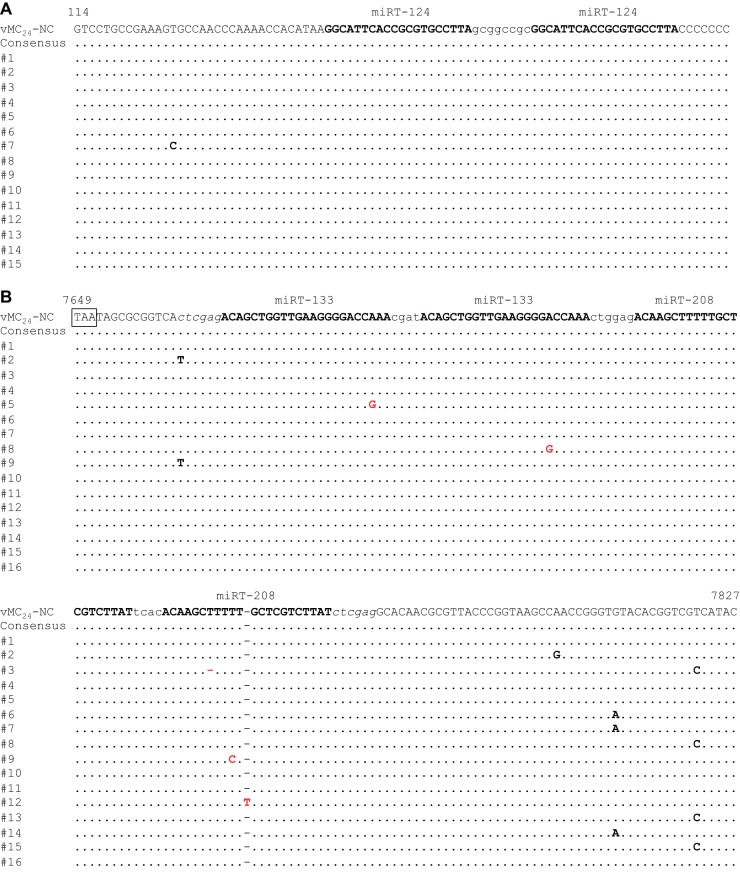

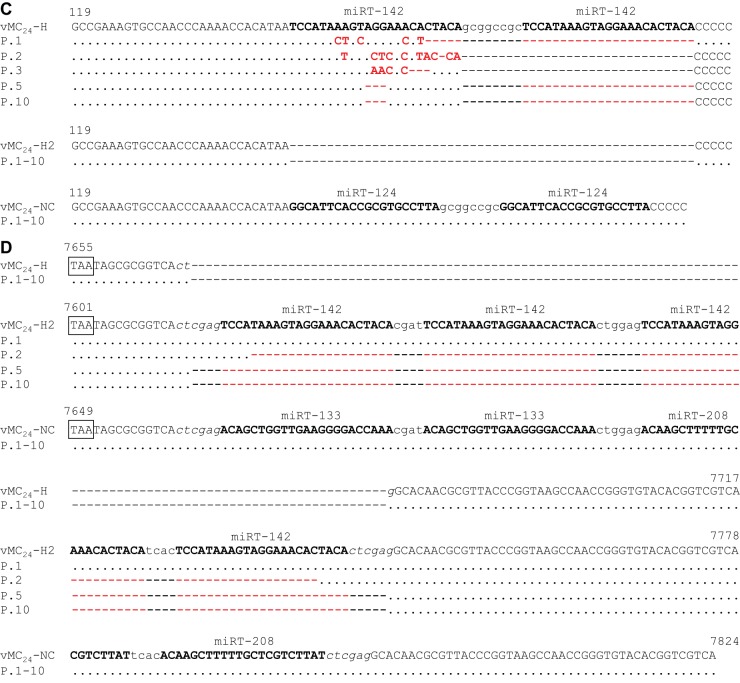
Genetic stability of miRNA target inserts *in vitro*. (A and B) vMC_24_-NC was serially passaged in H1HeLa cells using high-MOI inocula. (C and D) vMC_24_-H, -H2, and -NC were serially passaged in MPC-11 cells using high-MOI inocula. Viral RNA was isolated from clarified lysates of each passage stock, and the regions containing the miRTs were amplified via RT-PCR. PCR samples were directly sequenced to determine the consensus sequence. Amplicons from vMC_24_-NC passaged in H1HeLa cells also were cloned into an open cloning vector and individual clones sequenced. (A) Individual clone sequences of 5′ NCR amplicons from the tenth passage of vMC_24_-NC in H1HeLa cells. (B) Individual clone sequences of 3′ NCR amplicons from the tenth passage of vMC_24_-NC in H1HeLa cells. (C) Consensus sequences obtained from 5′ NCR amplicons of MPC-11 passaged viruses. (D) Consensus sequences obtained from 3′ NCR amplicons of MPC-11 passaged viruses. Identical bases are depicted as dots, and base mutations are shown in boldface. Mutations depicted in red denote mutations within an miRT sequence.

To determine whether the miRNA target sequences were genetically stable, vMC_24_-NC was serially passaged 10 times at high MOI in H1HeLa cells. The regions containing the miRNA target inserts were amplified from viral RNA isolated from the clarified lysate of passage ten. The PCR products were directly sequenced to determine the consensus sequence of the regions. The amplicons also were inserted into an open cloning vector and individual isolates sequenced. No changes were observed in either of the miR-124 target sequences in the 5′ NCR, and only one of the clones had a single base mutation in the region preceding the insert site (Fig. 4A). A higher frequency of single-base mutations was observed in the 3′ NCR (Fig. 4B). While no mutations were identified in the consensus sequence, single-base mutations in one of the four miRT inserts in the 3′ NCR were observed in four separate clones. Two of these mutations were in the seed sequence of the miRT insert.

To analyze the genetic stability of the miRT sequences under selective pressure, vMC_24_-H, -H2, and -NC were serially passaged 10 times at high MOI in MPC-11 cells, which express high levels of miR-142. The regions containing the miRT inserts were amplified from viral RNA isolated from the clarified lysates of passages one, two, three, five, and ten. The PCR products were directly sequenced to determine the consensus sequence of the regions (Fig. 4C and D). Similar to passaging in H1HeLa cells, no mutations were observed in the consensus sequences for either insert region of vMC_24_-NC. However, under selective pressure large deletions of the miRT sequences were observed in the consensus sequences of vMC_24_-H and vMC_24_-H2. Following the first passage, one of the miRTs in vMC_24_-H was deleted and several mutations were observed in the other. After five passages the consensus sequence maintained the deletion of one of the miRT sequences, with only a three-nucleotide deletion in the other. It is worth noting that the seed sequence in the remaining miRT sequence was intact. Following the first passage, no mutations were observed in the target sequences of vMC_24_-H2; however, all four inserts were completely deleted following passage five, and this was maintained through passage ten.

### vMC_24_-NC is a potent, nontoxic oncolytic virus.

**FIG 5:**
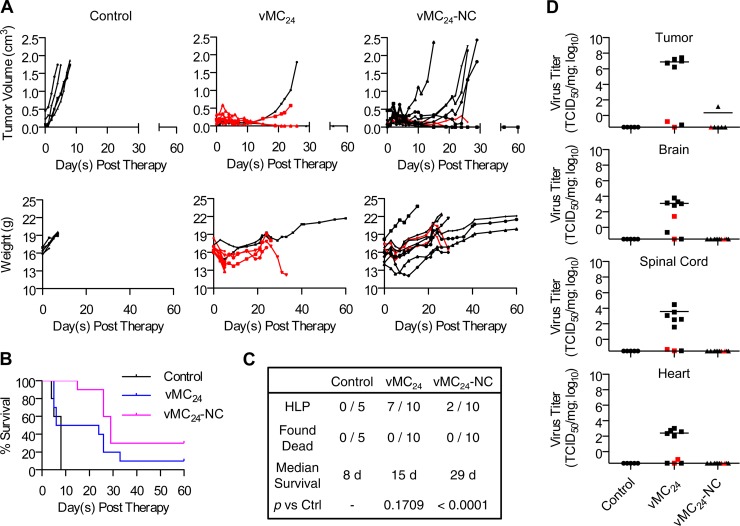
Therapeutic index of vMC_24_-NC is enhanced compared to that of vMC_24_. BALB/c mice bearing s.c. MPC-11 tumors were treated with a single i.t. injection of 1 × 10^6^ TCID_50_ vMC_24_ or vMC_24_-NC. (A, upper) Disease burden was monitored by calculating tumor volume versus time using repeat caliper measurements. (Lower) Weight was monitored for the duration of the experiment. Data highlighted in red indicate the animal developed HLP. (B) Overall survival of control Opti-MEM-treated (*n* = 5), vMC_24_-treated (*n* = 10), and vMC_24_-NC-treated (*n* = 10) mice was assessed using Kaplan-Meier survival curves. (C) Tabulation of causes of death/euthanasia aside from tumor volume in all treated mice, median survival, and significance of overall survival benefit in treated mice versus control (Ctrl) or vMC_24_-treated mice based on log-rank analyses. (D) Viral loads within tissues harvested at the time of euthanasia were determined. Data points in red represent animals that developed late-onset HLP.

The oncolytic efficacy of vMC_24_-NC compared to that of vMC_24_ was evaluated initially in immunocompetent BALB/c mice bearing s.c. MPC-11 tumors (Fig. 5). Mice received a single i.t. injection of vMC_24_ or vMC_24_-NC (1 × 10^6^ TCID_50_). Control (Opti-MEM)-treated mice displayed rapid tumor progression and were euthanized within 7 days following treatment. A transient loss in weight was observed uniformly in virus-treated mice initially following therapy. vMC_24_-treated mice exhibited rapid tumor regression, but 7 of 10 animals developed HLP, and 5 did so within 6 days posttreatment. Two mice developed HLP on days 24 and 26 posttherapy, but unlike the mice with rapid-onset HLP, they did not have significant infectious viral titers within the brain or spinal cord at the time of euthanasia (Fig. 5D). Additionally, tumor nodules were observed on the livers and spleens of these animals, suggesting that the HLP was caused by spinal cord compression due to tumor metastasis, a common feature of myeloma. Histological examination with hematoxylin and eosin (H&E) staining of tissues isolated from two mice that developed HLP on days 5 and 6 posttreatment revealed a focus of nonsuppurative inflammatory cells as well as a focus of hemorrhage in the white matter of the spinal cord in one of the mice (data not shown). In contrast to mice treated with vMC_24_, no early-onset HLP was observed in mice treated with vMC_24_-NC. Two of the 10 mice treated developed HLP on days 26 and 29 posttreatment. However, no infectious virus was recovered from the brain, spinal cord, heart, or muscle tissues of these mice (Fig. 5D), and tumor nodules were observed on their livers and spleens. Histological examination with H&E staining revealed a large area of neoplastic cells infiltrating the skeletal muscle and the possible aggregation of tumor cells in the meningeal membrane of the ventral cerebral hemisphere of one of these mice. However, no significant pathology was noted in the other mouse (data not shown). While not definitive, these results suggest non-virus-related mechanisms for HLP manifestation. All vMC_24_-NC-treated mice responded to therapy, with four having complete responses, four having partial responses, and two with delayed tumor growth. All mice with partial responses eventually succumbed to tumor burden; however, treatment with vMC_24_-NC significantly increased the overall mean survival compared to that of mice treated with untargeted vMC_24_ or Opti-MEM.

### Therapeutic efficacy is maintained following the systemic delivery of vMC_24_-NC.

**FIG 6:**
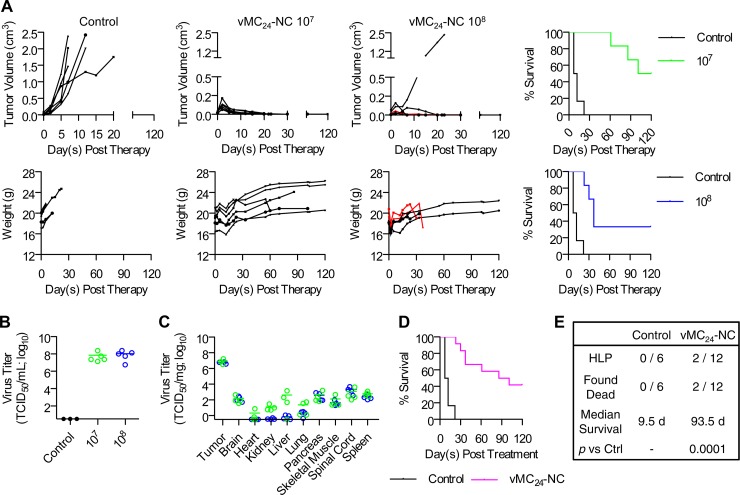
Therapeutic efficacy of vMC_24_-NC is maintained following systemic delivery. BALB/c mice bearing s.c. MPC-11 tumors were treated with a single i.v. injection via the tail vein of Opti-MEM control, 1 × 10^7^ TCID_50_ vMC_24_-NC, or 1 × 10^8^ TCID_50_ vMC_24_-NC. (A) Disease burden determined by calculating tumor volume versus time using repeat caliper measurements and weight of mice. Overall survival of mice treated with 1 × 10^7^ TCID_50_ vMC_24_ (*n* = 6) or 1 × 10^8^ TCID_50_ vMC_24_ (*n* = 6) compared to control Opti-MEM-treated mice (*n* = 6) was assessed using Kaplan-Meier survival curves. (B) Plasma viral loads in virus-treated mice were determined on day 2 after therapy. (C) Four days after virus treatment, four mice from each group were euthanized and viral loads within various tissues were determined. All tissues from control-treated mice were negative (data not shown). Note that one mouse per group did not have tumors at the time of harvest. (D) Overall survival of vMC_24_-NC-treated mice versus untreated animals was assessed using Kaplan-Meier survival curves. (E) Overall tabulation of the causes of death/euthanasia distinct from tumor burden in all treated mice, median survival, and significance of overall survival benefit in vMC_24_-NC-treated mice versus control mice as determined by log-rank statistical analysis.

In order to evaluate the therapeutic efficacy of vMC_24_-NC administered systemically, we treated immunocompetent BALB/c mice bearing s.c. MPC-11 tumors with a single i.v. dose of vMC_24_-NC at either 1 × 10^7^ or 1 × 10^8^ TCID_50_ via the tail vein (Fig. 6). In contrast to the rapid tumor progression observed in control Opti-MEM-treated mice, 11 of the 12 mice treated with vMC_24_-NC displayed the complete regression of the s.c. tumor. All of the virus-treated mice exhibited a transient weight loss initially following therapy (Fig. 6A). Among the mice treated with 1 × 10^7^ TCID_50_ vMC_24_-NC, one was found dead on day 61 posttreatment and one was euthanized on day 86 following the development of a scapular tumor with tumor nodules also noted on the liver postmortem. Among the mice treated with 1 × 10^8^ TCID_50_ vMC_24_-NC, one was found dead on day 37 and two other mice developed HLP on days 30 and 37. Similar to mice in the i.t.-treated cohorts, we attribute the late-onset HLP to metastasis, as multiple tumor nodules were observed on the livers and spleens of both mice postmortem. Additionally, no infectious virus was recovered from any tissues harvested at the time of euthanasia. Both dose levels of vMC_24_-NC significantly increased survival compared to that of control-treated mice and resulted in an overall survival rate of 42% at 120 days posttreatment (Fig. 6D). The biodistribution of the virus also was analyzed on day four posttreatment. Four mice per group were euthanized, and the viral titers in various tissues were determined (Fig. 6C). The highest viral loads were detected in the tumors, while low levels of virus were detected in tissues known to be susceptible to mengovirus infection, including the brain, spinal cord, and heart. Of note is that viral titers in mice treated with 1 × 10^8^ TCID_50_ vMC_24_-NC were lower in several of the tissues. Coupled with the overall survival comparison between the two virus-treated groups, these data suggest diminished viral distribution in the higher-dose cohort of mice.

### Genetic stability of miRNA target sequences *in vivo*.

**FIG 7:**
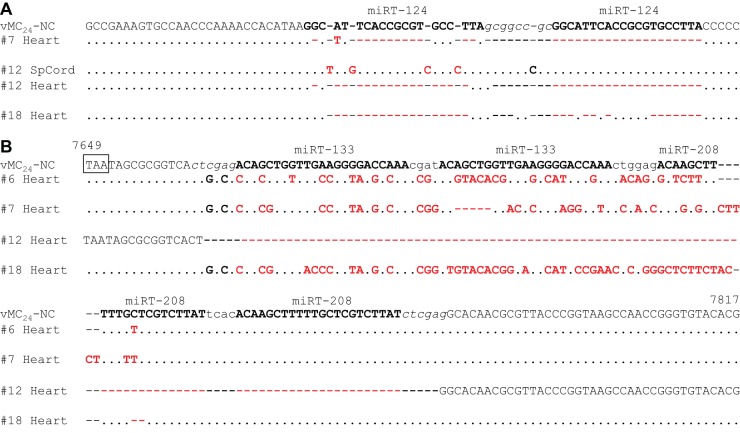
Genetic stability of miRNA target sequences following systemic delivery. Viral RNA was isolated from the clarified lysates of tumor, brain, spinal cord, heart, and spleen tissues isolated on day four posttherapy from BALB/c mice bearing s.c. MPC-11 tumors treated with a single i.v. dose of 1 × 10^7^ TCID_50_ (numbers 6 and 7) or 1 × 10^8^ TCID_50_ (numbers 12 and 18) vMC_24_-NC (*n* = 2 per group). Regions containing the miRTs were amplified via RT-PCR. PCR samples were directly sequenced to determine the consensus sequence. (A) Consensus sequences with mutations obtained from 5′ PCR amplicons. (B) Consensus sequences with mutations obtained from 3′ PCR amplicons. Identical bases are depicted as dots, and base mutations are shown in boldface. Mutations depicted in red denote mutations within an miRT sequence.

To determine whether the miRT sequences were genetically stable *in vivo*, viral RNA was isolated from the clarified lysates of tumor, brain, spinal cord, heart, and spleen tissues from four of the i.v.-treated mice euthanized on day four for biodistribution (*n* = 2 per group). The regions containing the miRTs were amplified and the PCR products directly sequenced to determine the consensus sequences. No mutations in any of the miRT sequences were observed in the tumor, brain, or spleen tissues from any of the mice. Three of the four mice had no mutations in the miR-124 target sequences isolated from the spinal cord. Virus isolated from the spinal cord of one mouse had several single-nucleotide insertions in the first miR-124 target sequence; however, no mutations were observed in the second. The amplification of miRT insert regions from the heart tissue proved difficult and required nested PCR. Following nested PCR, sequences with large deletions of the miR-124 target sequences were amplified in three of the four mice. One of the mice had no mutations in the miR-124 target sequences amplified from the heart tissue. A significant number of mutations were observed in the 133/208(2×) 3′ NCR insert in three out of four mice. The insert was completely deleted in one of the mice. Consensus sequences of tissues where mutations or deletions were observed are depicted in Fig. 7.

## DISCUSSION

Clinical trials evaluating oncolytic viruses have clearly demonstrated the potential of this therapeutic approach while simultaneously revealing important factors that may limit the success of the therapy (2–4, 43). These include the availability of relevant preclinical animal models, preexisting immune status of the patient, tolerability of effective doses, manufacturing feasibility, and virus extravasation. Based on these parameters, mengovirus is an ideal candidate for development as an oncolytic virotherapy. First, a range of cancers can be targeted due to the broad tropism of the virus, and the therapeutic efficacy and safety of the therapy can undergo extensive preclinical evaluation in relevant animal models with intact or suppressed immune systems. Second, the prevalence of mengovirus antibodies in humans is very low, circumventing limitations of preexisting immunity (15). Third, the entire viral genome can be inserted within a single plasmid and can be easily modified and virus particles manufactured. Additionally, mengovirus has a small particle diameter, facilitating extravasation from tumor blood vessels. Finally, mengovirus replicates very rapidly, resulting in high-level viremia, which may foster its distribution to disseminated sites of tumor growth and potentially minimize the dose required for therapeutic efficacy.

Mengoviruses with a truncated pCT (vMC_24_) or a deleted pCT are highly attenuated or avirulent, respectively, and both are promising vaccine candidates against EMCV-like viruses, resulting in no adverse reactions following vaccination (10, 15, 20, 21). It is feasible that less attenuated viruses will provide a more optimal balance between the oncolytic and immune phase of oncolytic virotherapy. Based on this prediction, we focused our initial analyses on the attenuated vMC_24_ versus the avirulent vMC_0_. However, it will be interesting to determine the potential of vMC_0_ as an oncolytic agent specifically in determining the role of the immune system in mengovirus therapy. Here, we show that vMC_24_ treatment of s.c. MPC-11 plasmacytomas resulted in partial or complete tumor regression; however, neurological and presumed cardiac toxicities are observed. Introducing vMC_24_ into a plasmacytoma provides a reservoir for viral replication and significant viremic potential, as shown in Fig. 6 for vMC_24_-NC. In addition to the increased dose necessary for therapeutic efficacy versus that needed for vaccination purposes, this environment likely is responsible for the increased virulence observed during our studies compared to previous studies examining the vaccine potential of vMC_24_. Additional studies examining the role that tumor size, initial viral distribution within a tumor, and the subsequent viremic levels play in therapeutic efficacy and virulence are needed. As the translation of this therapeutic virus to the clinical setting would require the elimination of these toxicities, the aim of this study was to enhance the safety profile of mengovirus using microRNA targeting technology.

MicroRNA targeting is a well-established mechanism for eliminating toxicities of oncolytic viruses (22, 23, 25, 44, 44–52). Few studies have demonstrated multitissue detargeting through the incorporation of universal miRTs or multiple tissue-specific targets. A recent report by Baertsch et al. focused on the incorporation of multiple tissue-specific target sequences at various positions within the measles virus genome as a preventative measure against toxicities that may arise during high-titer treatment (53). The simultaneous control of multiple unwanted tropisms by incorporating multiple miRTs into the viral genome also has been demonstrated for a telomerase-specific replication-competent adenovirus and a liver cancer-specific oncolytic herpes simplex virus (54, 55). Some studies also have used universally expressed Let-7 targets for broad control of oncolytic viruses, including vesicular stomatitis virus, poliovirus, and vaccinia virus (23, 45, 50). However, the miRNA expression profiles of cancer cells are heterogeneous among cancer types, patients, and even cells within a tumor. In the case of the Let-7 family of miRNAs, there are variations in expression levels among tissues and overlap in target sequence recognition among the various family members; therefore, even if one of the members is downregulated in a cancer cell the other family members can continue to target the virus, thereby reducing its efficacy. Our studies provide additional support for the simultaneous use of multiple miRNA targets to prevent tissue toxicities while maintaining oncolytic efficacy.

Sequences complementary to miR-142, miR-124, miR-125, miR-133, and miR-208 were successfully incorporated (individually or in combination) into the 5′ and 3′ NCRs of the vMC_24_ genome. The targeting specificity of the inserts was verified *in vitro*, both in the presence of exogenously delivered miRNA mimics and as naturally occurring levels of miR-125 and miR-142. Our results demonstrate that the engineered miRT inserts interact with their respective miRNAs without altering the replication kinetics of the virus in the absence of the cognate miRNA. Following i.c. injection, 5′ NCR insert viruses functioned as expected. In contrast, vMC_24_-H2 and vMC_24_-C had reduced viral titers in the brain and spinal cord, suggesting that these insertions can inhibit viral replication in the CNS. This inhibition of viral replication by the 3′ NCR insertions may be due to the presence of miR-142, miR-133, or miR-208 in certain cells or nonspecific effects of the insertions themselves. Mengovirus is predicted to have three conserved stem-loops in the 3′ NCR. The deletion of stem-loop I proved to be innocuous *in vitro*. However, when injected i.c. into Swiss mice, clinical signs of disease were either delayed or absent. Thus, this stem-loop was hypothesized to play a role in mengoviral neurovirulence, cellular tropism, or host immunoregulatory induction (41). While our 3′ insertion is not located within stem-loop I, it may have an effect analogous to that of the deletion of the loop based on its proximity to the region. We are currently evaluating whether replacement of stem-loop I with an miRT would further enhance the therapeutic index of this virus compared to that of the dual-targeted vMC_24_-NC virus.

miR-125b is enriched in hematopoietic stem cells, and macrophages express a high concentration relative to those of other immune cells and tissues (42). In contrast, miR-124 is absent or expressed at low levels in peripheral macrophages but is expressed within microglia (56, 57). Our single-step growth curve data corroborate these results with the exception of vMC_24_-N3C, which exhibited reduced replication kinetics in the RAW 264.7 macrophages. This decrease may be the result of low levels of miR-124 being expressed, binding of the repeated miR-124 target sequences by another miRNA with partial complementarity, and/or an insert-mediated inhibition of an important host-cell virus interaction. The manipulation of the length of the pCT has been shown to affect viral growth in macrophages; as the miRNA target sequences are directly adjacent to the tract, it is possible that an insert of a certain length or particular sequence can compromise the function of this sequence element. Because macrophages have important immune functions, we are conducting additional studies in order to determine the role macrophages play in mengovirus therapy and whether this is affected by the ability of the virus to replicate within these cells. Chaudhuri et al. showed that increased expression of miR-125b in macrophages enhances their response to IFN-γ, which may increase the rate of viral clearance diminishing therapeutic efficacy (42). Additionally, an upregulation of miR-125b enhanced by treatment with dexamethasone has been observed in multiple myeloma patients (58). Based on these observations, we focused our *in vivo* analyses on vMC_24_-NC; however, our *in vitro* data demonstrate the potential of engineering an arsenal of targeted mengovirus therapeutics tailored to specific miRNA expression profiles of different cancers. miR-134 is also highly expressed in the hippocampus, a major site of mengovirus pathology, and may serve as an additional alternative to miR-124 or miR-125 targets, expanding the versatility of this virotherapy. We have demonstrated previously that combining more than one miRT for a specific tissue can have additive affects in decreasing gene expression and therefore designed our 3′ NCR inserts as such (22). However, we did not see additive effects when miR-125 and miR-134 target sequences were combined for the purpose of enhancing the safety of vesicular stomatitis virus (44). Based on these results, we introduced our neuronal targets separately; however, it will be interesting to determine whether combining different neuronal targets will further enhance the safety profile of an miRNA-targeted mengovirus.

RNA viral genomes mutate rapidly due to the low fidelity of the viral polymerase, making the potential of escape mutants a concern when using miRNA targeting technology. The miRT sequences of vMC_24_-NC proved genetically stable *in vitro* following 10 serial passages in H1HeLa cells. Base mutations were observed in the 3′ NCR inserts at a frequency similar to those observed in the surrounding regions. However, when miRNA-targeted mengoviruses (vMC_24_-H and -H2) were serially passaged *in vitro* in the presence of the cognate miRNAs, large deletions within the miRTs were observed as early as passage one. However, viral RNA isolated from targeted tissues of mice treated systemically with vMC_24_-NC did not show this rapid selection. Mutations within the miRT inserts were observed only in the spinal cord of one mouse and in the heart tissues of all four mice. However, detection of these sequences required nested PCR, indicating an extremely low abundance of vRNA congruent with the low level of infectious virus recovery. This could be due to inherent differences in the selective pressure of specific miRNAs. It also could indicate that additional host factors reduce the rate at which escape mutants arise *in vivo*. While some mutations and deletions of the miRTs were observed in the targeted tissues, they did not result in viral toxicity. The combination of an attenuated virus backbone, tandem repeat of the targets, the use of multiple miRTs with overlapping tissue specificity, the potential for partial complementarity of different miRNAs, and the presence of an intact immune system decreases the potential that escape mutants will arise *in vivo* prior to viral clearance. Our results suggest that although escape mutations arise, they are insufficient to cause toxicity, likely due to the mounting of an antiviral immune response prior to their accumulation. Previous studies have demonstrated the genetic stability of the shortened pCT *in vitro* as well as following forced *in vivo* passaging in 4-week-old mice (10). Similar studies are under way in our laboratory to confirm whether inserting microRNA targets directly upstream of the pCT alters its stability *in vivo*.

vMC_24_-NC maintained oncolytic activity, did not cause lethal toxicities, and significantly increased survival in the MPC-11 myeloma model following i.t. or i.v. administration. Combining all cohorts of mice in these studies, 47% of mice treated with vMC_24_ succumbed to viral toxicity within 10 days following treatment, whereas no toxicity was observed in this time period in mice treated with vMC_24_-NC. Out of the 10 mice treated i.t. with vMC_24_-NC, four had complete regression of the s.c. tumor, four had partial regression, and two exhibited delayed tumor growth. Progressive disease eventually recurred in the six mice with delayed or partial response. Out of the 12 mice treated i.v. with either 1 × 10^7^ or 1 × 10^8^ TCID_50_ vMC_24_-NC, 11 had complete regression of the s.c. tumor, and the final mouse had delayed tumor growth. Two of the mice treated i.t. and two mice treated i.v. with 1 × 10^8^ TCID_50_ MC_24_-NC developed HLP between days 26 and 37 following virus administration. This could be due to virus reversion or progression of multiple myeloma disease. No infectious virus could be recovered from any of the tissues harvested from mice treated with either vMC_24_ or vMC_24_-NC that exhibited late-onset HLP. Additionally, postmortem examination of mice revealed consistent tumor metastases within these mice. Finally, while no definitive conclusions could be drawn from histological examination, evidence supporting a tumor-related development of HLP was noted in one of the mice treated with vMC_24_-NC. Overall, these results suggest that the development of HLP in these mice was not due to virus toxicity but rather the progression of myeloma disease burden.

Overall, systemic therapy with vMC_24_-NC resulted in a 42% survival rate in mice bearing s.c. MPC-11 plasmacytomas. The median survival of mice treated with a high dose (1 × 10^8^ TCID_50_) was significantly lower than that for mice treated with 1 × 10^7^ TCID_50_, and the reasoning for this is unclear. MPC-11 myeloma infiltration of liver and spleen has been shown following i.v. injection of tumor cells (59). On day four posttherapy, mice treated with the higher dose exhibited lower viral loads in several tissues, including the liver. Therefore, it is possible that viral distribution to additional tumor sites was diminished, allowing the more rapid progression of this aggressive cancer model. This difference also may be explained by the presence of defective interfering particles in the virus stock or their enhanced accumulation *in vivo* attenuating the overall persistence of infection.

The MPC-11 model proved semiresistant to mengovirus infection *in vitro*; however, potent oncolytic activity still was observed *in vivo*. It will be interesting to determine the therapeutic efficacy of vMC_24_-NC against a panel of cancer models *in vivo* that may prove even more susceptible. It also will be important in the future to determine the mechanisms behind variable susceptibilities among similar cancer cell lines. Previous studies have demonstrated the immunogenic potential of vMC_24_ as a vaccine candidate for EMCV-like viruses (10, 20, 21). As vMC_24_-NC virotherapy can be evaluated in immunocompetent animals, it will be interesting to examine its ability to cross-prime the immune system against tumor cells and determine the potential benefit of combining vMC_24_-NC with promising immunotherapies in order to enhance therapeutic efficacy. To this end, it also will be of interest to test the therapeutic potential of the pCT-deleted virus vMC_0_. Overall, our data indicate that mengovirus is a promising candidate for clinical translation as an oncolytic agent with the potential for broad clinical application or for vaccine development and that miRNA targeting can increase the therapeutic index of vMC_24_.
